# A Bayesian approach to modelling heterogeneous calcium responses in cell populations

**DOI:** 10.1371/journal.pcbi.1005794

**Published:** 2017-10-06

**Authors:** Agne Tilūnaitė, Wayne Croft, Noah Russell, Tomas C. Bellamy, Rüdiger Thul

**Affiliations:** 1 School of Mathematical Sciences, University of Nottingham, Nottingham, England, United Kingdom; 2 School of Life Sciences, University of Nottingham, Nottingham, England, United Kingdom; 3 Department of Electrical and Electronic Engineering, University of Nottingham, Nottingham, England, United Kingdom; Oxford, UNITED KINGDOM

## Abstract

Calcium responses have been observed as spikes of the whole-cell calcium concentration in numerous cell types and are essential for translating extracellular stimuli into cellular responses. While there are several suggestions for how this encoding is achieved, we still lack a comprehensive theory. To achieve this goal it is necessary to reliably predict the temporal evolution of calcium spike sequences for a given stimulus. Here, we propose a modelling framework that allows us to quantitatively describe the timing of calcium spikes. Using a Bayesian approach, we show that Gaussian processes model calcium spike rates with high fidelity and perform better than standard tools such as peri-stimulus time histograms and kernel smoothing. We employ our modelling concept to analyse calcium spike sequences from dynamically-stimulated HEK293T cells. Under these conditions, different cells often experience diverse stimulus time courses, which is a situation likely to occur *in vivo*. This single cell variability and the concomitant small number of calcium spikes per cell pose a significant modelling challenge, but we demonstrate that Gaussian processes can successfully describe calcium spike rates in these circumstances. Our results therefore pave the way towards a statistical description of heterogeneous calcium oscillations in a dynamic environment.

## Author summary

Upon stimulation a large number of cell types respond with transient increases of the intracellular calcium concentration, which often take the form of repetitive spikes. It is therefore believed that calcium spikes play a central role in cellular signal transduction. A critical feature of these calcium spikes is that they occur randomly, which raises the question of how we can predict the timing of calcium spikes. We here show that by using Bayesian ideas and concepts from stochastic processes, we can quantitatively compute the calcium spike rate for a given stimulus. Our analysis also demonstrates that traditional methods for spike rate estimation perform less favourably compared to a Bayesian approach when small numbers of cells are investigated. To test our methodology under conditions that closely mimic those experienced *in vivo* we challenged cells with agonist concentrations that vary both in space and time. We find that cells that experience similar stimulus profiles are described by similar calcium spike rates. This suggests that calcium spike rates may constitute a quantitative description of whole-cell calcium spiking that reflects both the randomness and the spatiotemporal organisation of the calcium signalling machinery.

## Introduction

Transient changes in the intracellular calcium (Ca^2+^) concentration have long been associated with the activation of plasma membrane receptors [1]. Since the seminal work by Woods et al. [2] linking the frequency of cytosolic Ca^2+^ oscillations in hepatocytes to the concentration of various hormones, both experimental and theoretical studies have provided compelling evidence for encoding extracellular stimuli into intracellular Ca^2+^ oscillations [3–14]. In whole-cell recordings, Ca^2+^ oscillations are usually observed as sequences of spikes of the intracellular Ca^2+^ concentration.

A prominent feature of Ca^2+^ spike sequences is that they are random. Ca^2+^ spikes only occur with some probability that generally changes over time. For example, there are distributions of inter-spike intervals (ISIs) for agonist induced Ca^2+^ oscillations in HEK293 cells and spontaneous Ca^2+^ oscillations in astrocytes, microglia and PLA cells instead of a single value [15]. When astrocytes are transiently stimulated with ATP three times, with a recovery period between stimuli, the observed Ca^2+^ spikes display any number of response patterns, from no spikes to three [16]. To elucidate the principles that govern the translation of extracellular cues into changes of the intracellular Ca^2+^ concentration therefore requires faithfully capturing the stochasticity in Ca^2+^ spike generation.

To date, the main modelling approach to investigate stochastic Ca^2+^ spike generation has been based on numerical solutions of differential equations, both ordinary and partial [11, 12, 15, 17–30]. In these studies, the randomness of Ca^2+^ spikes results from the stochastic behaviour of Ca^2+^ releasing channels, such as the inositol-1,4,5-trisphosphate (InsP_3_) receptor (InsP_3_R), and their interactions. The random dynamics of the InsP_3_R is then either described by coupling a Markov chain for the InsP_3_R to the differential equations, or by assuming a Langevin-type equation. All of these approaches require detailed models of the InsP_3_R (with often a considerable number of rate constants), and other assumptions such as the number of InsP_3_Rs per cluster and the spatial distribution of InsP_3_Rs, Ca^2+^ pumps and Ca^2+^ buffers in the case of partial differential equations. However, such mechanistic detail, which has been instrumental in advancing our understanding of Ca^2+^ spikes, often comes at considerable computational costs.

It might be therefore desirable to change the perspective from the mechanistic bottom-up approach to a top-down view, in which cellular Ca^2+^ spikes are described directly. The mathematical concept that has proven particularly useful for this endeavour is the theory of point processes [31]. Indeed, in [15] a time-dependent conditional rate for the generation of a Ca^2+^ spike was introduced, and its two parameters (a time scale and an amplitude) were determined from experiments on four different cell types. Subsequent work [32, 33] demonstrated in more detail that for constant stimulation, the time scale was cell type specific, while different cells of the same type could be distinguished by their amplitude.

These modelling approaches have not confronted the issue of the dynamic nature of cell stimulation that occurs under physiological conditions. Cells in vivo experience a complex and dynamically-changing environment, where signals frequently arrive in a time-varying manner—such as transient release of neurotransmitters or oscillations in the concentration of circulating hormones. Diffusion of messengers through tissue (such as away from a blood vessel) also introduce spatial variation in signal strength, meaning that cell populations encounter a complex spatiotemporal pattern of stimulation. An efficient mechanism for modelling Ca^2+^ responses in such a heterogeneous population has not yet been devised. Our approach offers a solution to this issue and is based on combining point processes with Bayesian inference.

Bayesian concepts have been used with great success across various disciplines (see e.g. [34] for an overview). One advantage of utilising Bayesian ideas is that model parameters can be effectively constrained by observed data and can be estimated in a controlled fashion. For example, each set of parameter values comes with its own probability that informs us about how likely this set represents the observed data. In the past, the combination of Bayesian inference and point processes has been successfully applied to action potential spike trains in neurons [35, 36], but to our knowledge, this is the first time that Ca^2+^ spike sequences have been analysed in this way. While we can draw on these previous results, the substantial differences between action potential spike trains and Ca^2+^ spike sequences (e.g. the number of spikes per train or the time scales of spikes) have required significant attention.

A particular characteristic of Ca^2+^ spikes is that their generation depends on the cellular Ca^2+^ spike history and hence is non-Markovian. This results from both the stochastic nature of Ca^2+^ spike formation as well as the dynamic variation in the cellular signalling micro-environment. To model such history dependence, we follow a concept introduced in [37], which effectively turns a non-Markovian Ca^2+^ spike sequence into a Markovian one. This is achieved with the help of a so-called intensity function *x*(*t*), for which we provide a definition and more details in the Materials and Methods section. Since the intensity function is directly inferred from individual Ca^2+^ spike sequences, it is specific to each cell. This results from two facts. Firstly, Ca^2+^ spikes are shaped by the cellular composition of the Ca^2+^ signalling toolkit, i.e. the expression levels and spatial arrangement of Ca^2+^ channels, pumps, transporters and buffers. Secondly, each cell experiences a different signalling micro-environment as illustrated above. Autocrine and paracrine signalling modify the original signal further. In addition, any time-dependence of *x*(*t*) that originates from a time varying signal is compounded by dynamic changes of the Ca^2+^ signalling apparatus, such as Ca^2+^ store refilling, adaptation or desensitisation.

In the present paper we demonstrate how to estimate *x*(*t*) in the presence of such single cell variability. This will allow us to make progress in two main directions. Firstly, one of the most highly discussed questions in computational biology is concerned with scaling dynamics from single cells to tissue. Taking into account our current understanding of intracellular Ca^2+^ signalling, this will require the simulation of spatially extended single cells driven by fluctuating Ca^2+^ releasing channels. The computational costs for such studies are extraordinary, and how to sample the associated high dimensional parameter space remains an open challenge. On the other hand, generating Ca^2+^ spike sequences from an intensity function is computationally cheap and hence puts researchers in an advantageous position to obtain high quality statistical insights into tissue level dynamics. Secondly, while our approach is statistical, the Ca^2+^ ISI distribution that we determine as part of estimating *x*(*t*) has direct mechanistic interpretations. This may further our understanding of how exactly Ca^2+^ spikes emerge at the single cell level from the orchestrated action of the Ca^2+^ signalling toolkit. For instance, one line of argument suggests that Ca^2+^ spikes result from the co-ordinated interplay of a certain number of Ca^2+^ puffs. Our statistical analysis may provide quantitative estimates for this.

## Results

### ISI statistics of Ca^2+^ spikes

**Fig 1:**
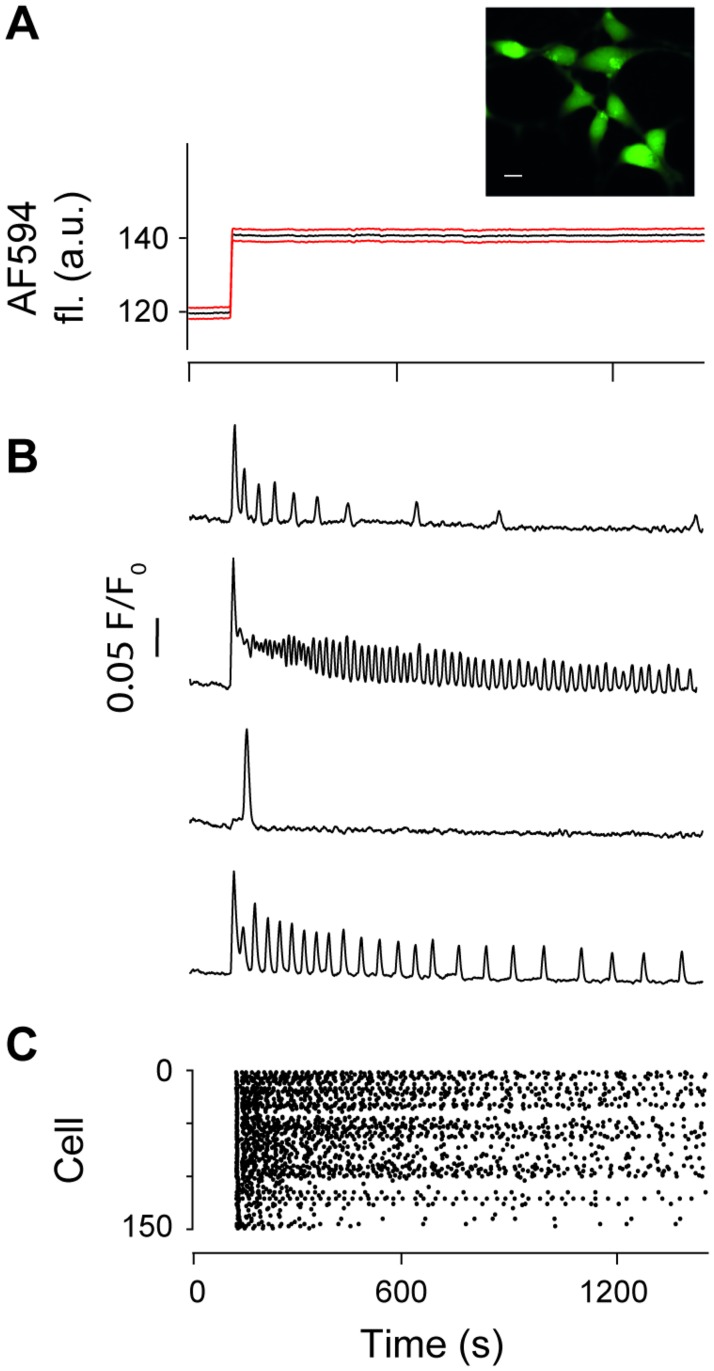
Heterogeneous Ca^2+^ responses. Cellular Ca^2+^ responses to a step-change in agonist concentration. (A) Cultured HEK293T cells were seeded in micro-channels, loaded with Fluo-5F AM (inset, scale bar: 10 *μ*M) and perfused with imaging buffer. A step-change in flow rates between solution containing carbachol (100 *μ*M) plus AF594 (2 nM) and buffer alone results in delivery of agonist (and AF594 indicator) to stimulate the cells. Mean (black) ± SEM (red) AF594 fluorescence from ROIs centred on cells within the microfluidics channel, showing onset of agonist exposure. (B) Normalised Fluo-5F fluorescence intensity traces show typical Ca^2+^ responses from four representative cells. (C) Raster plot of the pattern of Ca^2+^ spikes detected in the entire cell population. The Ca^2+^ spike sequences follow no particular order.

A main motivation for our study is the need to model the heterogeneity of Ca^2+^ responses observed in cell populations in a computationally efficient manner. To illustrate the problem, the results in Fig 1 show HEK293T cells challenged with a solution containing 100 *μ*M carbachol. Fig 1B illustrates the large variability of Ca^2+^ responses observed between different cells even when the stimulus is stationary. While some cells only detect the onset of the stimulus (third trace), other cells exhibit Ca^2+^ spikes during the entire stimulation period. However, the spike characteristics vary significantly. Some cells show irregular Ca^2+^ spikes (first trace), while other cells settle into an almost regular pattern (fourth trace). It is also worth noting that the frequency of Ca^2+^ spikes spans a considerable range (cf. first and second trace) and that all cells display a decrease in peak amplitude, which is indicative of adaptation. Fig 1C and S1 Video provide further evidence for the large heterogeneity in the timing of Ca^2+^ spikes for a constant stimulus.

The first step in developing a top-down model that reproduces this heterogeneity is to determine the probability distribution that most accurately describes recorded ISIs. Throughout this study, Ca^2+^ spikes are treated as all-or-nothing events and any information on the width or amplitude of a Ca^2+^ spike is excluded. The reason why we can introduce an ISI distribution and hence treat successive ISIs as independent—instead of trying to fit a time-dependent ISI distribution—comes from the introduction of the intensity function *x*(*t*). We tested three possible candidate ISI statistics (see Materials and methods for details): an inhomogeneous Poisson (IP), an inhomogeneous Gamma (IG) and an inhomogeneous inverse Gaussian (IIG) distribution. The IP distribution often serves as starting point for analysing spiking behaviour as it is the most basic statistical distribution. While the parsimony of the IP distribution has undoubtedly helped in establishing a large body of mathematical results, real world data often exhibit more complex statistics. The IG distribution provides a natural extension of the IP distribution, in that it contains the IP distribution as a special case: putting *γ* = 1 in (5) recovers (10). The shape parameter *γ* endows the IG distribution with more flexibility, which has proven fruitful in numerous applications. Conceptually, spikes in general and Ca^2+^ spikes in particular have been described as first passage events [38]. One of the most fundamental models, which contains positive drift and random motion only, gives rise to the IIG distribution.

**Fig 2:**
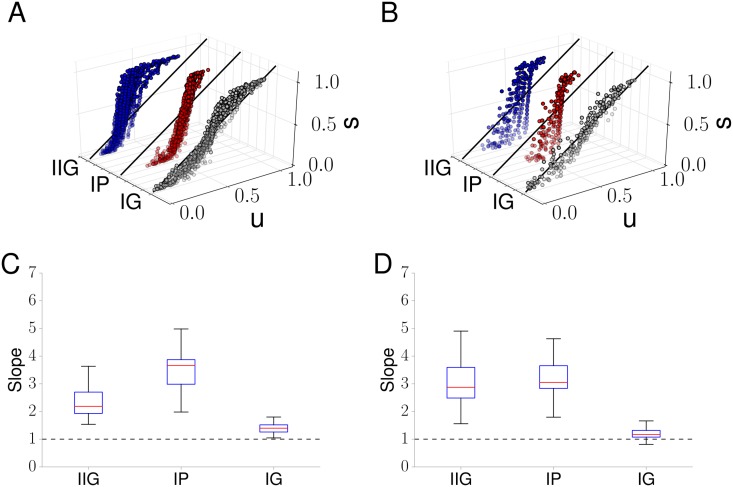
Determining Ca^2+^ spike ISI statistics. Kolmogorov-Smirnov plots for Ca^2+^ spike sequences in HEK293T cells stimulated with (A) 100*μ*M and (B) 10*μ*M carbachol when the ISI statistics is assumed to be an IIG (blue), IP (red) and IG (grey) distribution. Box and whisker plots summarising the Kolmogorov-Smirnov plots for (C) 100*μ*M and (D) 10*μ*M carbachol stimulation for the IP, IIG and IG models. The results in (A) and (C) are based on the data shown in Fig 1. In (C) and (D), the box extends from the first quartile (Q1) to the third quartile (Q3) with the red line at the median. The lower whisker corresponds to the smallest data point that is bigger than Q1−1.5×IQR, while the upper whisker extends to the largest value that is smaller than Q3+1.5×IQR, where IQR denotes the interquartile range Q3-Q1. We used 42 cells in (A), (C) and 21 cells in (B), (D).

To ascertain which ISI distribution describes the results in Fig 1 best, we transformed the recorded ISIs using the time rescaling theorem and analysed the results in a Kolmogorov-Smirnov plot (see Material and methods). The Kolmogorov-Smirnov plot allows the visual inspection of whether two probability distributions are the same by plotting their respective cumulative distribution functions against one another. If the two distributions are identical, the cumulative distributions functions coincide and plotting one against the other results in a straight line with slope 1. Fig 2A shows results for the Kolmogorov-Smirnov plot for the data taken from Fig 1. Here, *u* corresponds to the cumulative distribution of the transformed data, which depends on the chosen ISI distribution, while *s* stems from a theoretical prediction based on the time rescaling theorem (see Materials and methods). If we have identified the correct ISI distribution, data points should cluster around a straight line with a 45° slope. Note that in our analysis, each cell was treated individually and no data amalgamation took place.

Results for the IP and IIG clearly deviate from a line with slope 1, while the data for the IG exhibit much less deviation. This suggests that an IG, but not an IP or IIG, better describes the ISI statistics of Ca^2+^ spikes. The box plots in Fig 2C provide further quantitative evidence. They demonstrate that the slopes obtained from individual cells are concentrated close to 1 and exhibit a significantly smaller variability for the IG compared to the IP and IIG. To further corroborate these findings, we analysed data from cells exposed to a lower concentration of carbachol (10*μ*M). Fig 2B and 2D illustrate that again the IG distribution captures the ISI statistics most closely. In addition to Kolmogorov-Smirnov plots, we also tested our data with a quantile-quantile plot (see S1 Fig and Materials and methods). As with the Kolmogorov-Smirnov plot the correct ISI distribution leads to data points that accumulate around the 45° line. Panels A and B in S1 Fig show that this is the case for the IG, but not for the IP and IIG, hence confirming our results from the Kolmogorov-Smirnov plot. The box plots in panels C and D of S1 Fig show that again the slopes obtained from single cells for the IG model are much closer to one and exhibit less variability than those for the IP and IIG. Taken together, these results indicate that the patterns of Ca^2+^ spikes observed across the population of cells can most accurately be reproduced with an IG distribution. This approach will therefore be used as the basis of our analysis.

It is worth noting that when testing the different ISI distributions, we also obtain an estimate for the intensity function *x*(*t*). As illustrated by Eq (11), the probability for a specific ISI depends on *x*(*t*). Therefore, all ISI distributions in this study have to be understood as being conditioned on *x*(*t*). In the next section, we show how to estimate *x*(*t*) from measured Ca^2+^ spike sequences.

### Performance of Ca^2+^ spike rate estimation

**Fig 3:**
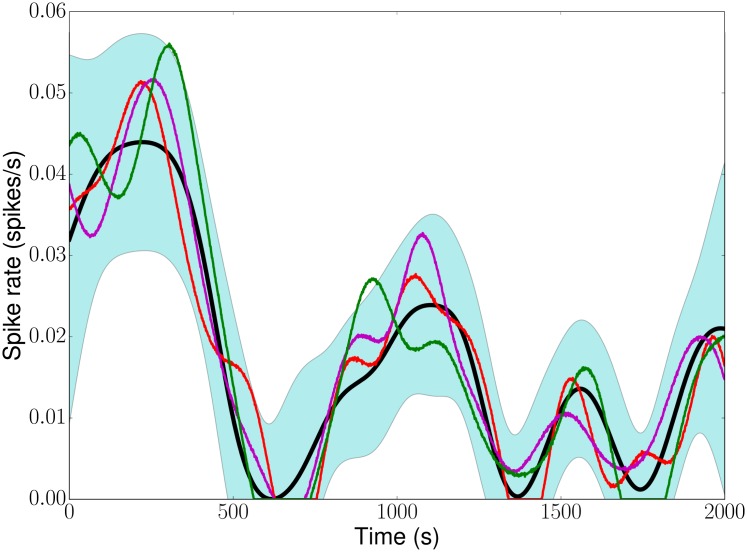
Gaussian process. Three realisations of a GP (red, green, purple) around a time-dependent mean (black line). The blue area delineates the 95% confidence interval. Note the changes in width, which reflect a time-dependent standard deviation.

In addition to the ISI distribution, we also need to know the intensity function *x*(*t*) to fully describe Ca^2+^ spike sequences. In the following, we will use three different approaches to estimate *x*(*t*): peri-stimulus time histograms (PSTHs), kernel smoothing (KS), and Gaussian processes (GPs) combined with Bayesian inference. An illustration of a GP is shown in Fig 3. In contrast to a deterministic curve, a GP generates infinitely many curves (3 possible candidates are shown), and the *statistics* of these curves is the organising principle. At each time point, the values of a GP are Gaussian distributed, and the mean and standard deviation can change over time. To guarantee a controlled comparison between PSTHs, KS and GPs, we will fix an intensity function, generate surrogate Ca^2+^ spike sequences from it and then estimate how well the above methods recapture the original intensity function (see Materials and methods for details).

Before proceeding it is worth noting that the intensity function that we need to estimate is identical to the Ca^2+^ spike rate that we find from using either PSTHs or KS [36]. In other words, if we can obtain a high quality estimate for the Ca^2+^ spike rate from either PSTHs or KS, we have a very good estimate for *x*(*t*). However, this usually requires a large number of Ca^2+^ spike sequences, which is an issue that we will address below. Since we can identify the Ca^2+^ spike rate with the intensity function, we will use both terms interchangeably. Note, however, that this Ca^2+^ spike rate is different from the conditional intensity function defined in Eq (15), which is often used in generating spike trains.

**Fig 4:**
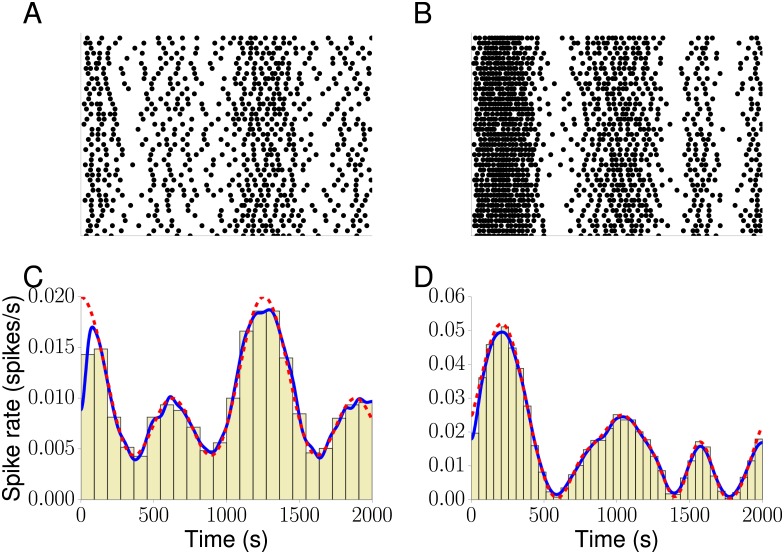
Ca^2+^ spike sequences. (A-B) Raster plots of 40 out of 100 Ca^2+^ spike sequences simulated from *x*_det_ and *x*_GP_, respectively. (C-D) Estimations of *x*_det_ and *x*_GP_ from all generated Ca^2+^ spike sequences based on a PSTH (beige) and KS (solid blue). The true values of *x*_det_ and *x*_GP_ are shown as a dashed red line. Ca^2+^ spike sequences were generated using inverse sampling. Parameter values are (A,C) *γ* = 5.9 and (B,D) *μ* = 2.1, *σ*_*f*_ = 1.5, *κ* = 0.5 and *γ* = 6.2.

A major objective of this model is to reproduce Ca^2+^ signalling patterns during complex stimulation conditions. For example, physiological patterns of hormone or neurotransmitter release are time-varying, rather than the step-changes used in typical experiments (such as Fig 1). We therefore tested the performance of candidates for *x*(*t*) for reproducing dynamically-changing signals—specifically sinusoidal oscillations. As a first choice, we considered a regularly oscillating intensity function *x*_det_(*t*) = 0.5 cos(*t*) + 0.5 cos(0.5*t*) + 1. Fig 4A shows Ca^2+^ spike sequences generated from *x*_det_, and Fig 4C reveals that both PSTH and KS capture the original intensity function very well.

By using a specific functional form for *x*(*t*) as in *x*_det_(*t*), we make strong assumptions about the intensity function. A more flexible and versatile approach is based on GPs. In Fig 4B we plot Ca^2+^ spike sequences generated from one *x*_GP_ candidate, while Fig 4D depicts the estimation of *x*_GP_ from a PSTH and KS. As with *x*_det_ we find very good agreement between the original and estimated Ca^2+^ spike rate.

This strategy supposes that all Ca^2+^ spike sequences can be combined into a single large dataset, but in a physiological context this will not always be true. To be a more useful tool, the model should be able to simulate the diversity of responses expected in a more complex environment, where the stimulus varies in both space and time. Under these circumstances, the number of cells that receive an equivalent stimulus would be more limited, and so it is important to assess how the number of spike sequences available for parameter estimation affects the accuracy of predicting the Ca^2+^ spike rate. Consequently, we randomly picked groups of 1, 2, 4 and 7 Ca^2+^ spike sequences and computed the Ca^2+^ spike rate based on a GP and KS.

**Fig 5:**
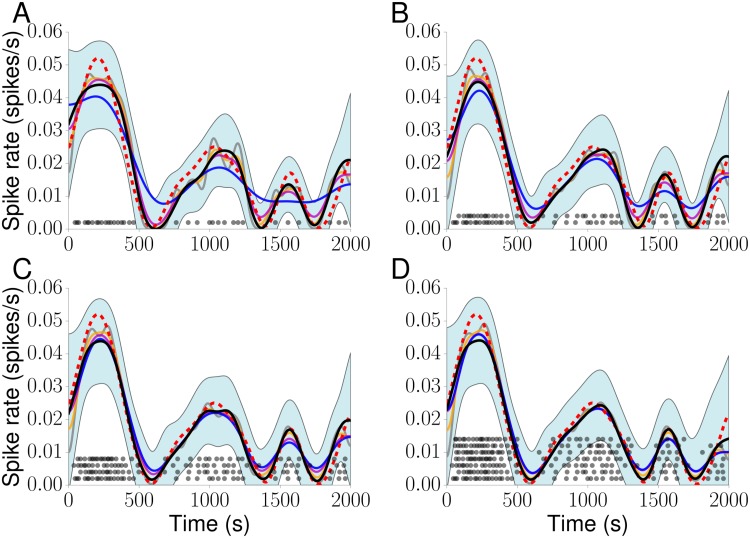
Ca^2+^ spike rate estimation. Spike rate estimations for data shown in Fig 4B for 1 (A), 2 (B), 4 (C) and 7 (D) randomly chosen Ca^2+^ spike sequences (black dots) using KS with *σ* = 35 (grey), 52 (orange), 70 (purple), and $\hat{\sigma }$ (blue). The black line denotes results from a GP(black). The dotted red line indicates the original Ca^2+^ spike rate. The light blue areas delineate the 95% GP estimation confidence interval.

In Fig 5 we show results for *x*_GP_. Since the Ca^2+^ spike rate estimation obtained from KS depends on the bandwidths *σ* (see Eq (17)), we employed different *σ* values. For a single Ca^2+^ spike sequence (Fig 5A) the estimated Ca^2+^ spike rates differ visibly from the theoretical one, and the smallest bandwidth leads to spurious oscillations. As we increase the number of Ca^2+^ spike sequences the estimated Ca^2+^ spike rates capture the true Ca^2+^ spike rate more faithfully. The light blue area in each panel delineates the 95% confidence interval, which we obtain as a by-product from the GP optimisation. Overall, all estimates lie within this confidence interval except the one for $\hat{\sigma }$ in Fig 5A (see Materials and methods for the definition of $\hat{\sigma }$). We obtain similar results for *x*_det_(*t*) as illustrated with S2 Fig.

**Fig 6:**
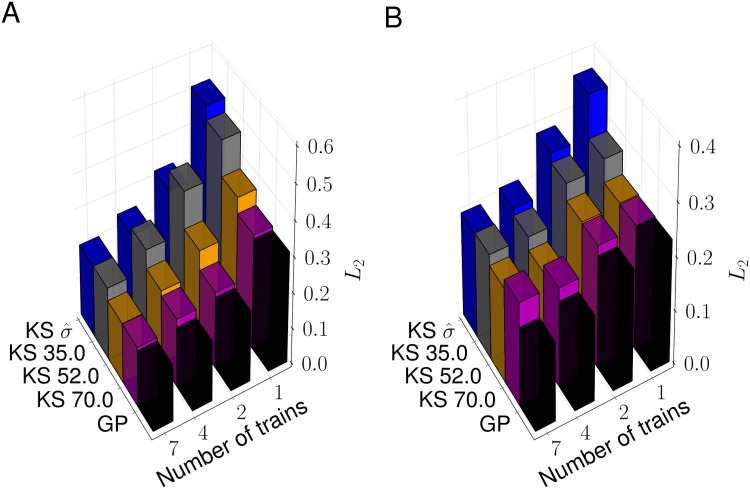
Comparison of Ca^2+^ spike rate estimations. Normalised *L*_2_ norm for the estimates of *x*_det_ (A) and *x*_GP_ (B) from the results in Fig 5 and S2 Fig.

We quantified the accuracy of predicting the Ca^2+^ spike rate by computing the normalised *L*_2_ norm of the difference between the known and estimated Ca^2+^ spike rate (see Materials and methods). Fig 6 shows that for a given method, the *L*_2_ norm decreases as we increase the number of Ca^2+^ spike sequences, which corresponds to better predictions. When we fix the number of Ca^2+^ spike sequences, GPs yield a better estimate. The improvement is particularly evident when comparing the Ca^2+^ spike rate estimation based on $\hat{\sigma }$ at small numbers of Ca^2+^ spike sequences. We further tested that our results did not depend on the particular choice of Ca^2+^ spike sequences nor on the details of the surrogate generator. For the latter, we compared three different approaches: inverse sampling, a Bernoulli process and time rescaling. We generated a number of Ca^2+^ spike sequences with each method and then estimated the Ca^2+^ spike rate using the same methods as in Fig 6, i.e. KS with different bandwidths and GPs. S3 Fig shows box plots of the normalised *L*_2_ norm between the estimated and the true Ca^2+^ spike rate *x*_det_. We find that for all three methods, the normalised *L*_2_ norm is generally smallest for GP estimates. To test the statistical significance of this result, we computed the corresponding *p*-values as shown in S1 Table using the non-parametric Mann-Whitney test. Based on the common assumption that a finding is statistically significant if *p* < 0.05, estimates using GPs perform statistically better than KS since the largest *p* value was 0.0375. We repeated the analysis for *x*_GP_ and report the normalised *L*_2_ norm in S4 Fig. While the GP performs clearly better than KS with bandwidths of $\hat{\sigma }$ and 35, the distributional results for bandwidths of 52 and 70 look similar to those of the GP. This is also confirmed by the *p*-values shown in S1 Table, where some exceed the threshold of 0.05. This indicates that KS can approach the performance of GPs. However, since there are no *a priori* estimates for this optimal bandwidths for a given scenario, GPs provide the more robust estimation method.

### Estimation of Ca^2+^ spike rates in HEK293T cells

The results so far provide strong evidence that an intensity function derived from a GP allows accurate prediction of Ca^2+^ spike patterns even when the estimate is based on small numbers of Ca^2+^ spike sequences. We next applied our Bayesian approach to a more complex experimental system, designed to reproduce some of the stimulus heterogeneity expected *in vivo*.

**Fig 7:**
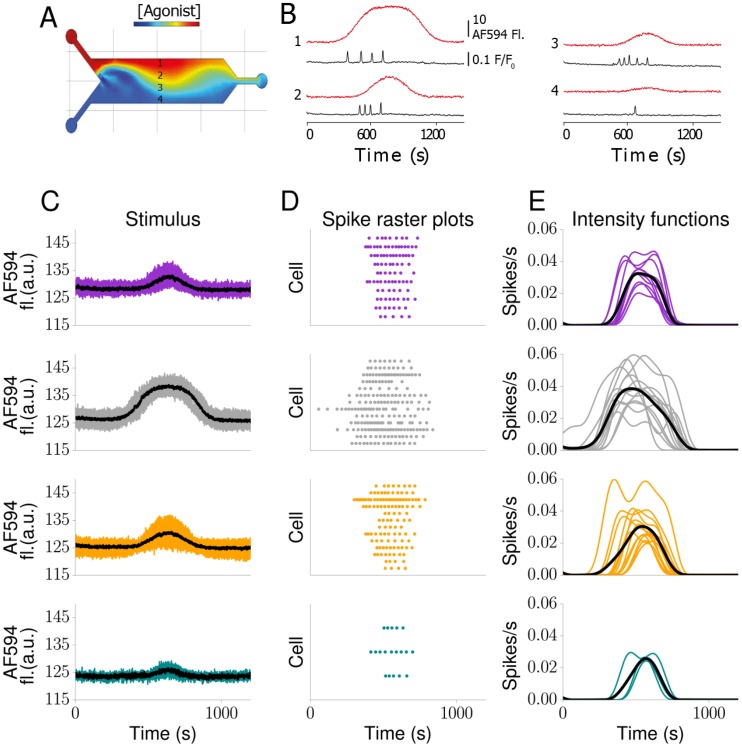
Dynamically stimulated HEK293T cells. (A) Simulation of complex concentration surface generated inside the microfluidics chamber after transiently varying relative flow rates of agonist and buffer input streams (COMSOL Multiphysics, COMSOL Ltd, Cambridge, UK). (B) Agonist concentration (indicated by AF594 fluorescence; red lines) and normalised Fluo-5F fluorescence intensity traces (black lines) from ROIs centred on 4 cells across the width of the channel upon applying a single sine-wave stimulation regime. Stimulus fluorescence (C), raster plots (D) and GP Ca^2+^ spike rate estimations (E) for each cluster in Fig 8. Black lines denote the mean stimulus (C) and the mean Ca^2+^ spike rate (E) in each group, respectively.

We used a microfluidics chamber to deliver sinusoidal changes in carbachol concentration to HEK293T cells (see Materials and methods). The concentration of carbachol varied in both space and time. Fig 7A shows a complex concentration surface throughout the chamber at a fixed time point and illustrates how the sharp interface between high and low agonist concentration on the left side of the chamber widens as the flow progresses through the chamber. In Fig 7B we plot agonist concentration time courses sampled at four positions along the transverse direction of the microfluidics chamber, which demonstrates the stimulation heterogeneity that cells experience depending on their position within the chamber. We also include the corresponding Ca^2+^ spike sequences, which again display significant variability. The goal of this experiment was to generate an environment in which a population of cells is exposed to agonist in a manner that varies with the cells’ distance from the stimulus source and with a dynamic mechanism of delivery (by analogy to a circulating hormone diffusing from a blood vessel to underlying cells, for example).

**Fig 8:**
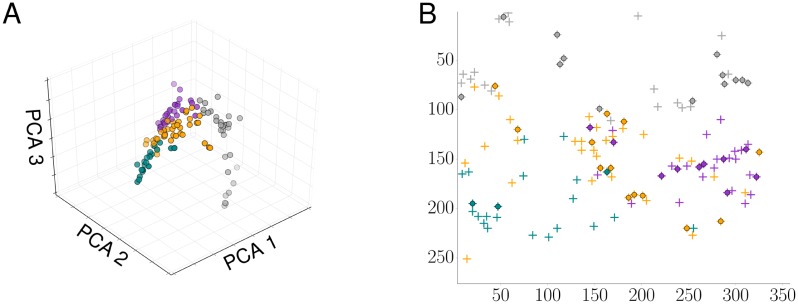
Clustering of dynamic stimuli and cell positions. (A) Weights of the three leading principal components of the stimulus data for the experiment of Fig 7. (B) Position of cells in the microfluidics chamber are shown by a plus sign. Cells that were included in the analysis, i.e. those that spiked more than 10 times, are identified by a circle. Stimuli (and hence cell positions) belonging to the same group as identified by the *k*-means algorithm are coloured identically and with the same colour as in Fig 7.

This scenario is the context in which a Bayesian framework is most useful, as it can predict spiking Ca^2+^ responses to a complex but physiologically meaningful stimulus profile. In Fig 7C–7E, we show clustered stimulus curves, the corresponding Ca^2+^ spike sequences and the estimated intensity functions, respectively. We grouped cells that experienced similar agonist concentration profiles to allow for a meaningful comparison of the resultant intensity functions. To determine how similar stimulus traces are, we computed the weights of the three leading principal components (see Materials and methods). Results are shown in Fig 8, where we employed *k*-means [39] to detect possible clusters. Data points that belong to the same group are plotted in the same colour, and these colours correspond to those used in Fig 7C–7E. Overall, 4 distinctive groups represented the data best, containing 10, 13, 13 and 3 cells, respectively. It is worth noting that while clustering was performed on the stimuli time courses the Ca^2+^ spike sequences show a consistent pattern in that they are generally more similar within a given group than between groups.

The black lines in Fig 7C and 7E denote the mean stimulus and mean intensity function, respectively. We observed that the intensity functions broadly mirror the global behaviour of the stimulus. The mean intensity function for all responses showed a single peak with a similar time course to the stimulus. The amplitude of stimulus and intensity function are also well matched. However, there are also observable differences. For example, individual cells showed intensity functions with a more complex time course than the mean (such as multiple peaks). Furthermore, while the mean stimulus was symmetrical, the mean intensity functions could exhibit notable asymmetries. For example, for weak stimuli, the rising phase of the intensity function may be markedly slower than the falling phase (Fig 7C and 7E; yellow and green traces). This most likely reflects the excitable character of intracellular Ca^2+^ signalling [30, 40]. For weaker stimulation, it takes longer to reach the threshold for generating a Ca^2+^ spike, hence the intensity function grows more slowly. The quicker decrease results from the stimulus dropping below the Ca^2+^ spike generating threshold quickly after reaching its maximum, hence prohibiting further Ca^2+^ spikes. The faster increase of the intensity function for stronger stimuli (grey traces) lends further support for this interpretation, as the Ca^2+^ spike generating threshold is reached more quickly.

**Fig 9:**
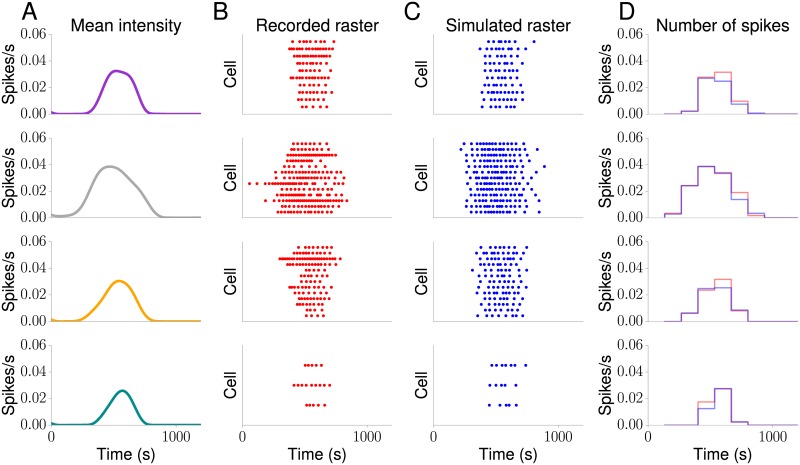
Surrogate Ca^2+^ spike sequences from experimentally determined Ca^2+^ spike rates. (A) Mean intensity functions as shown in Fig 7E. The colours correspond to the ones used in Fig 7. (B) Raster plot of recorded Ca^2+^ spike sequences as shown in Fig 7D. (C) Surrogate Ca^2+^ spike sequences generated from the mean intensity function depicted in (A). (D) Histograms of recorded and simulated Ca^2+^ spike sequences.

To illustrate the value of the mean intensity functions shown in Fig 7E, we generated surrogate Ca^2+^ spike sequences from them using the IG distribution and plotted them in Fig 9C. For ease of comparison, we also show the measured Ca^2+^ spike sequences in Fig 9B. We first note that the simulated Ca^2+^ spike sequences resemble the measured ones. This is also confirmed by the histrograms in Fig 9D, which exhibit large overlaps between the experimental and theoretical Ca^2+^ spike sequences. To quantify how similar the two histograms are, we computed the histogram distance given by

$$\begin{array}{c} H\left(R,S\right)=\frac{\sum_{i}\min(R_{i},S_{i})}{\max(\sum_{i}\;R_{i},\sum_{i}\;S_{i})}\;, \end{array}$$ (1)

where R_*i*_ and *S*_*i*_ denote the histogram count in the *i*th bin of the recorded and simulated data, respectively. The histogram distance is bounded between 0 and 1, and the closer it is to 1 the more similar the histograms are. The histograms coincide if *H* = 1. We found that *H* = 0.86, 0.94, 0.91 and 0.89 (from top to bottom), which confirms our visual inspection that the histograms vary little between recorded and simulated Ca^2+^ spike sequences. Moreover, Ca^2+^ spike sequences generated from one intensity function exhibit a certain degree of heterogeneity, which is consistent with our experimental findings. Taken together, these results show that without explicitly including any information about the stimulus into the estimation of the intensity functions, our approach yields intensity functions that reflect the characteristics of the stimulus and that are consistent with experimentally recorded Ca^2+^ spike sequences.

## Discussion

A key task for all cell types is to faithfully respond to external stimuli. For signalling pathways that rely on the dynamics of the intracellular Ca^2+^ concentration, sequences of Ca^2+^ spikes have long been recognised as the likeliest encoding mechanism of extracellular cues. Detailed numerical simulations have provided a mechanistic understanding of how cells generate Ca^2+^ spike sequences and have demonstrated the emergence of ISI fluctuations from subcellular processes such as Ca^2+^ puffs. Conceptually, such modelling falls into the class of bottom-up approaches. In the present study, we have adopted a top-down perspective in that we have developed a modelling framework that directly describes the stochastic timing of Ca^2+^ spikes at the cellular level. Importantly, our data-driven approach implicitly accounts for subcellular details through the introduction of the intensity function *x*(*t*).

Our modelling approach is based on the idea of representing Ca^2+^ spike sequences as realisations of a point-process. In contrast to earlier applications of this concept, consecutive Ca^2+^ spikes in the current study do not necessarily exhibit the same statistics, i.e. the ISI distribution may become time-dependent. This is a direct consequence of the time-varying stimulation. However, through appropriately transforming the times of Ca^2+^ spikes and by using the time-dependent intensity function *x*(*t*), it is possible to describe Ca^2+^ spike ISIs with one distribution for the entire Ca^2+^ spike sequence.

We therefore began our investigation by testing different ISI distributions: inhomogeneous Poisson (IP), inhomogeneous Gamma (IG) and inhomogeneous inverse Gaussian (IIG). The IP process is a common choice as it is the simplest stochastic process and the only one for which the conditional intensity function *q*(*t*|*y*_*k*_, *x*) coincides with the intensity function *x*(*t*) (see Eq (15)). This greatly facilitates the mathematical analysis, which can draw on a large body of already established results. However, the IP process often fails to describe experimental spike trains, see e.g. Barbieri et al. [37]. We therefore turned to the more general IG process, which includes the IP process as a special case. In addition, we employed the IIG distribution. Our results in Fig 2 show that the IG distribution captures experimental ISIs very well, whereas both the IP and IIG distribution poorly represent the Ca^2+^ spike data. While these results all pertain to whole cell Ca^2+^ spikes, they also shed further light on the details of the subcellular processes that generate these Ca^2+^ spikes. The IG distribution with shape parameter *γ* results from sampling an IP process every *γ*th spike. In other words, the ISI distribution for an IG is the same as if one measured the ISIs for *γ* successive spikes generated from an IP and then added all *γ* ISIs up to obtain a single ISI. Interestingly, one proposed mechanism for the generation of Ca^2+^ spikes is wave nucleation, where a critical number of Ca^2+^ puffs has to occur [19, 41]. Under the assumption that Ca^2+^ puffs are described by an IP process, the above arguments entails that *γ* can be linked to the number of Ca^2+^ puffs to trigger a Ca^2+^ spike. From a more general perspective Ca^2+^ wave nucleation can be considered as a first passage time problem, since we are interested in the first time that a critical number of Ca^2+^ puffs occurs. Importantly, the concept of first passage times is also at the heart of Ca^2+^ puff generation [42–44]. Assuming a continuous representation for Ca^2+^ spike generation, one of the simplest first passage time problems describes Brownian motion with positive drift to reach a fixed level for the first time [45]. The associated probability distribution is the IIG distribution, which we chose as our third candidate. The failure of the IIG distribution to capture the behaviour of Ca^2+^ spikes may point towards more complex subcellular dynamics than random motion and positive drift.

We used two different tests to determine the most likely ISI distribution, a quantile-quantile plot and a Kolmogorov-Smirnov plot. If we correctly identified the ISI distribution that is consistent with experimental data, the measured ISIs can be transformed to obey an exponential ISI distribution. Both the quantile-quantile plot and the Kolmogorov-Smirnov plot interrogate how closely the transformed ISIs are described by an exponential distribution. The fact that the two tests focus on different aspects of the distribution [46, 47] and that both identified the IG as the most plausible ISI distribution provides strong support for our findings. While our analysis suggests that the IG distribution describes the measured data best, we cannot rule out that other distributions that we have not tested, e.g. a log-normal distribution or a generalised exponential distribution, might yield equally good or even better results. If future work reveals another probability distribution than the IG distribution that is consistent with the data, the discussion will turn towards the stochastic processes that generate these distributions and how they reflect the physiology of Ca^2+^ signalling. We will touch on this point later in the discussion.

Our modelling framework rests on two pillars: an ISI distribution and a Ca^2+^ spike rate. While the ISI distribution may shine light on potential mechanisms that generate Ca^2+^ spikes, the Ca^2+^ spike rate encodes the speed of Ca^2+^ spike formation. To estimate the Ca^2+^ spike rate, we used the fact that it coincides with the intensity function *x*(*t*). Given that there are numerous ways to generally estimate intensity functions from experimental data, we tested three approaches with particular emphasis on Ca^2+^ spikes: Bayesian inference with GPs, KS and PSTHs. As Fig 4 illustrates, both KS and PSTHs yield excellent results when we have a large number of Ca^2+^ spike sequences that are all generated from the same intensity function. One of the reasons for these good estimates is that by pooling all Ca^2+^ spike sequences, the statistics become effectively Poissonian [31, 48] and that in this case an optimal bandwidth for KS [49, 50] and an optimal bin size for the PSTH [49, 51] is known. However, no *a priori* estimates for a bandwidth or bin size exist when only a small number of Ca^2+^ spike sequences with a few spikes each is available. As Fig 6 shows the relative error in estimating the true Ca^2+^ spike rate strongly depends on the bandwidth when Ca^2+^ spikes are generated from an IG process and estimates are based on only a few Ca^2+^ spike sequences. This severely limits the use of KS and PSTHs in estimating Ca^2+^ spike rates from experimental recordings since firstly the ISI statistics are not Poissonian, and secondly combining Ca^2+^ spikes from a large number of cells might not be possible as we will discuss below. We therefore turned to Bayesian inference using GPs. Importantly, the Bayesian approach already works for a single Ca^2+^ spike sequence. By using a prior distribution *p*(*θ*) for the hyperparameters *θ*, i.e. the parameters that describe the shape of the ISI distribution and that control the behaviour of the GP, we explicitly represent the uncertainty associated with each hyperparameter. This alleviates the need for fixing parameter values prior to the estimation of the Ca^2+^ spike rate as is the case for PSTHs and KS. Fig 6 shows that GPs yield better results than KS. Moreover, we also obtain confidence intervals from the GP optimisation (Fig 5), which allows us to judge the quality of the Ca^2+^ spike rate estimation *a posteriori*.

Spike rates are often estimated from pooled data. This practice is well founded if cells generate spikes with the same mechanism. When cells are stimulated, this approach also assumes that each cell experiences the same stimulus time course. For ligand-dependent signalling pathways, the last condition is usually met experimentally by exposing cells to a constant stimulus. However, this might not be the situation *in vivo*. We therefore used a microfluidics chamber to challenge HEK293T cells with a time-varying concentration of carbachol. As Fig 7 illustrates, different cells experience different concentration profiles of carbachol. Importantly, when computing the average over all stimuli time courses, not a single cell experiences this specific stimulation. To identify cells that are stimulated in a similar manner and hence can be compared with each other, we computed the weights of the three leading principal components of each stimulus time course, and then used a *k*-means algorithm. We then determined the most likely Ca^2+^ spike rate for each cell and computed the mean Ca^2+^ spike rate for cells within a given group (Fig 7). Overall, there is substantial variability in the Ca^2+^ spike rates with respect to their mean. This results from the variation in the Ca^2+^ spike sequences, which is illustrated by a comparison between the first and the third group. This variability in the Ca^2+^ spike rates leads to intriguing questions. On the one hand, the fluctuations could arise from the intrinsic stochasticity of Ca^2+^ spike generation. It is known that cells challenged repeatedly with the same stimulus (and allowing for recovery between successive stimulation) respond randomly (see e.g. [16]). Hence, the observed Ca^2+^ spike sequences constitute a sample of the possible cellular responses given a particular stimulus. On the other hand, the variability could stem from the composition of the Ca^2+^ signalling apparatus in each cell. At the subcellular level, a Ca^2+^ spike often corresponds to a travelling Ca^2+^ wave that is shaped by Ca^2+^ release from intracellular storage compartments such as the endoplasmic reticulum and Ca^2+^ sequestration by Ca^2+^ pumps [30, 52–54]. The spatial arrangement of Ca^2+^ releasing channels, Ca^2+^ pumps and Ca^2+^ buffers strongly affects Ca^2+^ waves and therefore Ca^2+^ spikes [11]. Given that even genetically identical cells express different numbers of the components of the Ca^2+^ signalling toolkit and arrange them in different spatial patterns, the variability in the Ca^2+^ spike rates could reflect single cell variability at the molecular level. The two sources for the variability of Ca^2+^ spike rates are not mutually exclusive, and a mixture of both is most likely to occur *in vivo*.

To bring order to such disparate Ca^2+^ spike sequences, recent studies have shown that when cells are challenged with a constant stimulus, a linear relationship exists between the mean and the standard deviation of ISIs [12, 15]. The slope of this relationship was shown to be robust to interventions at the molecular level (blocking Ca^2+^ pumps, energising Ca^2+^ release channels) as well as being cell type and agonist specific. A theoretical analysis revealed that the slope could be determined by a recovery timescale from global cellular inhibition after a Ca^2+^ spike. Therefore, one expects that each cell type and each agonist can be characterised by this timescale. In the present study, each cell possesses its own intensity function *x*(*t*). The organising principle that will lead to a cell type and stimulation specific description of Ca^2+^ spiking is given by the parameter values that describe the *statistics* of *x*(*t*), i.e. the hyperparameters of the GP. Put differently, for a given cell type stimulated with a specific agonist and application protocol, we expect *one* set of parameter values for the GP. Heterogeneous cell responses then originate from different realisations of the GP. To illustrate this concept, assume for the time being that the intensity function is constant. In a population, there will be a spread of Ca^2+^ spiking behaviour, with some cells only generating a few Ca^2+^ spikes, while others exhibit high Ca^2+^ spiking activity. Consequently, the intensity function for slow spiking cells is low, while it is large for high frequency cells. For independent cells, as is the case in this study, it is reasonable to assume that the values of the intensity function are normally distributed. This is equivalent to saying that the intensity function of each cell is a realisation of a GP. The assumption of one set of hyperparameters for a given experiment and cell type will allow us to quantitatively compare different experiments and answer questions such as how different cell types respond to the same stimulus, or how different stimuli shape Ca^2+^ spike sequences in a given cell type. We expect that similar responses will be mirrored in small differences between hyperparameter sets.

The last point raises the question of how transferable results are from one cell type to another and from one stimulation scenario to another. In addition, the ultimate goal of the modelling framework presented here is to apply it to physiologically relevant tissues. For example, gap junctional coupling between cells, or paracrine signalling, could potentially influence population heterogeneity. This would require an extension of the modelling framework towards network dynamics, which may be a formidable challenge, as predicting the network behaviour from single node dynamics is nontrivial, let alone inferring single cell dynamics from within a connected tissue.

### Conclusion

In this work we have developed a mathematical framework to quantitatively describe the heterogeneous timing of Ca^2+^ spikes in a cell population subject to time-varying stimulation. At the heart of this new approach is the use of Bayesian inference to determine the most likely intensity function and hence the most likely Ca^2+^ spike rate for a given stimulus. As part of this estimation process, we found that the statistics of Ca^2+^ ISIs are best captured by an IG distribution. Importantly, knowledge of the intensity function and the ISI statistics suffices to completely describe Ca^2+^ spiking. Since generating Ca^2+^ spike sequences from an ISI distribution and intensity function is computationally significantly cheaper than solving partial differential equations for cellular Ca^2+^ transport, this approach is ideally suited for numerically studying large numbers of cells.

The estimation of inhomogeneous single cell behaviour also puts us in an ideal position to ascertain whether or not there is signal processing at the cell population level. Indeed, numerous examples exist where the average population behaviour is not shared by any cell (see e.g. [55]). These incongruous dynamics also warrant investigations into population invariances, where cell populations respond consistently in the same manner, albeit with completely heterogeneous single cell behaviour [56, 57]. By reliably estimating single cell Ca^2+^ dynamics, the present study provides a stepping stone towards answering these questions for intracellular Ca^2+^ signalling.

## Materials and methods

### Intensity function and Ca^2+^ ISI probability distributions

We here follow the exposition in [37] for the definition of the intensity function. Assume that Ca^2+^ spikes occur at times *y*_1_ < *y*_2_ < … < *y*_*N*_. Let *p*(*v*) denote the probability density for a general renewal process on *v* ∈ (0, ∞), i.e. *p*(*v*)d*v* is the probability for an event in [*v*, *v* + d*v*], and subsequent events are independent. For *y*_*a*_ > 0, let *y* correspond to a time variable on (*y*_*a*_, ∞) and *X* be a one-to-one mapping *X*(*y*) = *v* of (*y*_*a*_, ∞) to (0, ∞). Conservation of probability then entails that

$$\begin{array}{c} p\left(y\right)=|\frac{dv}{dy}|\;p\left(v\right)=|X^{\prime }\left(y\right)|\;p\left(X\left(y\right)\right)\;. \end{array}$$ (2)

In other words, the probability density for a Ca^2+^ spike at *y*_*i*_ can be computed from the renewal probability density *p* if we know the mapping *X*. A convenient form of *X* is

$$\begin{array}{c} X\left(y\right)=\int_{y_{a}}^{y}x\left(u\right)du\;, \end{array}$$ (3)

which satisfies the conditions above and where *x* is called the intensity function, which is the object that we need to estimate. Eq (3) can be interpreted as rescaling the original time *y* such that Ca^2+^ ISIs become independent and identically distributed in the new time [58]. Given two subsequent Ca^2+^ spikes times *y*_*i*−1_ and *y*_*i*_ in the original time, the ISI in the new time is

$$\begin{array}{c} X\left(y_{i-1},y_{i}\right)=\int_{y_{i-1}}^{y_{i}}x\left(u\right)du\;. \end{array}$$ (4)

Since it is only through the introduction of the intensity function *x*(*t*) that Ca^2+^ ISIs become Markov, we introduce the notation *p*(*y*_*i*_, *y*_*i*−1_|*x*), which corresponds to the ISI probability density given *x*(*t*). Note that formally the conditional ISI probability density is defined as the joint conditional probability density for spikes at *y*_*i*_ and *y*_*i*−1_ (and hence no spike in [*y*_*i*−1_, *y*_*i*_]) given an intensity function *x*(*t*). We will employ three different choices for the ISI probability density: an inhomogeneous Gamma distribution

$$\begin{array}{c} p(y_{i},y_{i-1}|x)=\frac{\gamma x\left(y_{i}\right)}{\Gamma \left(\gamma \right)}{\left[\gamma X\left(y_{i-1},y_{i}\right)\right]}^{\gamma -1}{\text{e}}^{-\gamma X\left(y_{i-1},y_{i}\right)}, \end{array}$$ (5)

where *γ* > 0 denotes the shape parameter and Γ is the Gamma function; an inhomogeneous inverse Gaussian distribution

$$\begin{array}{c} p(y_{i},y_{i-1}|x)=\frac{x\left(y_{i}\right)}{\sqrt{2\pi X^{3}\left(y_{i-1},y_{i}\right)}}\text{exp}\left\{-\frac{{\left(X\left(y_{i-1},y_{i}\right)-\alpha \right)}^{2}}{2\alpha^{2}X\left(y_{i-1},y_{i}\right)}\right\}, \end{array}$$ (6)

where *α* > 0 is the location parameter; and an IP distribution

$$\begin{array}{c} p\left(y_{i},y_{i-1}|x\right)=x\left(y_{i}\right)e^{-X(y_{i-1},y_{i})}\;. \end{array}$$ (7)

### Bayesian inference

The time-dependent intensity function *x*(*t*) is modelled as a Gaussian Process (GP) [34, 59]. A GP is uniquely defined by its mean *μ*(*t*) and covariance function Σ(*t*_1_, *t*_2_). While there are many possible choices for Σ [34, 60], we employ the widely used squared exponential (SE) kernel

$$\begin{array}{c} \Sigma \left(t_{1},t_{2}\right)=\sigma_{f}^{2}e^{-\frac{\kappa {\left(t_{1}-t_{2}\right)}^{2}}{2}}+\delta \left(t_{1}-t_{2}\right)\sigma_{v}^{2}\;, \end{array}$$ (8)

where *κ* measures the smoothness of the GP and *σ*_*f*_ controls its variance. The last term allows us to model additional noise sources. We originally included $\sigma_{v}^{2}$ as a hyperparameter in the optimisation. However, we consistently found small values for $\sigma_{v}^{2}$ and hence decided to fix it at a presentative value of $\sigma_{v}^{2}=10^{-4}$. We collect the spike times in a sequence of *N* Ca^2+^ spikes in a vector **y** = {*y*_1_, …, *y*_*N*_}. For consistency, we set *y*_0_ = 0. Through the introduction of an intensity function *x*(*t*), the joint probability density for a spike sequence **y** given *x*(*t*) factorises and reads as [36]

$$\begin{array}{c} p\left(y|x\right)=p_{1}\left(y_{1}|x\right)p_{T}\left(T,y_{N}|x\right)\prod_{i=2}^{N}\;p\left(y_{i},y_{i-1}|x\right)\;. \end{array}$$ (9)

Here, *p*_1_(*y*_1_|*x*) represents the conditional probability density of finding the first spike at time *y*_1_. We also take into account that the observation time *T* usually exceeds the last spike time through the term *p*_*T*_(*T*, *y*_*N*_|*x*), which denotes the conditional probability that no spike occurs after *y*_*N*_. The statistics for *p*_1_ and *p*_*T*_ are often based on an inhomogeneous Poisson (IP) process, i.e.

$$\begin{array}{c} p_{1}\left(y_{1}|x\right)=x\left(y_{1}\right)e^{-X(0,y_{1})}\;,\;p_{T}\left(T,y_{N}|x\right)=e^{-X(y_{N},T)}\;, \end{array}$$ (10)

where *X* is given by Eq (3).

For practical purposes, we discretise time with a time step Δ such that *T* = *n*Δ [36]. When working with experimental spike trains, we set Δ equal to the inverse of the recording frame rate. A spiking time *y*_*i*_ can then be expressed as *y*_*i*_ = *l*_*i*_ Δ for an appropriate $l_{i}\in N$. By setting *x*_*i*_ = *x*(*i*Δ) and using Eq (9) with e.g Eq (5), we obtain the probability density for a spike sequence for the inhomogeneous Gamma distribution as

$$\begin{array}{c} p(y|x,\theta )=x_{l_{1}}{\text{e}}^{-{\hat{X}}_{0,1}}{\text{e}}^{-{\hat{X}}_{N,n}}\prod_{i=2}^{N}\frac{\gamma x_{l_{i}}}{\Gamma \left(\gamma \right)}{\left[\gamma {\hat{X}}_{i-1,i}\right]}^{\gamma -1}{\text{e}}^{-\gamma {\hat{X}}_{i-1,i}}, \end{array}$$ (11)

where ${\hat{X}}_{i,j}=\Delta \sum_{k=l_{i}}^{l_{j}}x_{k}$ and *l*_0_ = 0, *l*_*n*_ = *n*. By introducing *θ* on the left hand side, we make explicit the dependence of the probability density on the hyperparameters *θ*, which in this case are *θ* = {*γ*, *κ*, *σ*_*f*_}.

The most probable intensity function x*(*t*) given a spike train **y** is determined by x* = argmax_*x*≥0_ *p*(*x*|**y**). Under the assumption that the nodal value x* is close to its mean, we have

$$\begin{array}{c} x^{*}\approx \int x_{\theta }^{*}p\left(\theta |y\right)d\theta =\frac{1}{Z}\int x_{\theta }^{*}F\left(y,x_{\theta }^{*},\theta \right)d\theta \;, \end{array}$$ (12)

where

$$\begin{array}{c} x_{\theta }^{*}=\underset{x\geq 0}{\mathrm{argmax}}p\left(x|y,\theta \right)=\underset{x\geq 0}{\mathrm{argmax}}p\left(y|x,\theta \right)p\left(x|\theta \right)\;. \end{array}$$ (13)

To evaluate the first integral in Eq (12) we note that

$$\begin{array}{c} p\left(\theta |y\right)=\frac{p(\theta )}{p(y)}\int p\left(y|x,\theta \right)p\left(x|\theta \right)dx=\frac{F(y,x_{\theta }^{*},\theta )}{p(y)}\;, \end{array}$$ (14)

with $F\left(y,x_{\theta }^{*},\theta \right)=p\left(\theta \right)p\left(y|x_{\theta }^{*},\theta \right)p\left(x_{\theta }^{*}|\theta \right)/\sqrt{\left|\Lambda^{*}+\Sigma^{-1}\right|}$ and $\Lambda^{*}=-L_{x}^{2}\log p\left(y|x_{\theta }^{*},\theta \right)$, where we used Laplace’s approximation for the integral as shown in S1 Appendix. We further introduced the notation $L_{x}^{2}f$ to denote the Hessian of *f* with respect to *x*(*t*) and $Z=\int F\left(y,x_{\theta }^{*},\theta \right)d\theta =p\left(y\right)/{\left(2\pi \right)}^{n/2}$.

### Time rescaling, quantile-quantile and Kolmogorov-Smirnov plots

Let *q*(*t*|*y*_*k*_, *x*), *t* > *y*_*k*_ denote the conditional intensity function, i.e. *q*(*t*|*y*_*k*_, *x*)d*t* is the probability for a spike in [*t*, *t* + d*t*] given an intensity function *x*(*t*) and the last spike at *y*_*k*_. We can express *q*(*t*|*y*_*k*_, *x*) in terms of the ISI probability density as [31]

$$\begin{array}{c} q\left(t|y_{k},x\right)=\frac{p(t,y_{k}|x)}{1-\int_{y_{k}}^{t}p\left(s,y_{k}|x\right)ds}\;. \end{array}$$ (15)

The time rescaling theorem then states that the rescaled ISIs [31, 47, 61, 62]

$$\begin{array}{c} \tau_{k}=\int_{y_{k-1}}^{y_{k}}q\left(s|y_{k-1},x\right)ds\;, \end{array}$$ (16)

are independent and identically distributed exponential random variables with mean one if **y** is a realisation from a point process with conditional intensity function *q*(*t*|*y*_*k*_, *x*).

Suppose there are *K* rescaled ISIs. For a quantile-quantile plot [46], we order the *τ*_*k*_ in ascending order giving rise to the new ISIs ${\tilde{\tau }}_{n}$. We then plot the quantiles of the distribution of the ${\tilde{\tau }}_{n}$ against the quantiles of an exponential distribution with unit rate, which are given by ${\hat{\tau }}_{n}=-\ln\left(1-s_{n}\right)$ with *s*_*n*_ = (*n* − 0.5)/*K*.

For the Kolmogorov-Smirnov, plot [62], we define the random variable $u_{k}=1-e^{-\tau_{k}}$ and then plot the ordered set of the *u*_*k*_ against the cumulative distribution function of the uniform distribution, i.e. *F*(*x*) = *x* for 0 ≤ *x* ≤ 1, sampled at *s*_*n*_.

### Kernel smoothing and *L*_2_ norm

The Ca^2+^ spike rate is estimated from *m* spike sequences via kernel smoothing (KS) through [49, 63]

$$\begin{array}{c} r=\frac{1}{m}\sum_{j=1}^{m}\sum_{i=1}^{N_{j}}\;f\left(t-y_{i}^{j},\sigma \right)\;, \end{array}$$ (17)

where $y_{i}^{j}$ denotes the *i*th spike time in the *j*th Ca^2+^ spike sequence **y**_*j*_, and *N*_*j*_ is the total number of spikes in **y**_*j*_. The function *f* represents the kernel, and we chose a Gaussian of the form

$$\begin{array}{c} f\left(t,\sigma \right)=\frac{1}{\sqrt{2\pi \sigma^{2}}}\exp(-\frac{t^{2}}{2\sigma^{2}})\;. \end{array}$$ (18)

The parameter *σ* is referred to as the bandwidth of the kernel. In case we work with a large number of independent Ca^2+^ spike sequences **y**_*j*_, we can use an optimal bandwith [49, 50]. To evaluate how well a given method (e.g. Bayesian inference or KS) approximates the true Ca^2+^ spike rate used to generate surrogate data, we evaluated the normalised *L*_2_ norm as

$$\begin{array}{c} L_{2}={\left[\int_{0}^{t}{\left(\hat{r}\left(t\right)-\tilde{r}\left(t\right)\right)}^{2}\text{d}t\right]}^{1/2}\;{\left[\int_{0}^{t}\tilde{r}\left(t\right)\text{d}t\right]}^{-1}, \end{array}$$ (19)

where $\tilde{r}$ and $\hat{r}$ denote the known and estimated Ca^2+^ spike rate, respectively.

### Principal component analysis and clustering

We arrange the stimuli experienced by individual cells in a matrix *X* such that each row corresponds to a single stimulus time course. We then compute the singular value decomposition of *X*, i.e. *X* = *U*Σ*V*^*t*^, where *t* denotes transposition. The columns of *V* correspond to the eigenvectors of *X*^*t*^*X*, and Σ is a diagonal matrix that holds the singular values of *X*. The weights of the principal components of the stimuli time courses are the rows of *XV* = *U*Σ.

The *k*-means algorithm requires the number *k* of clusters as input and then determines the members of each cluster by minimising the error function [64]

$$\begin{array}{c} E=\sum_{i=1}^{k}\sum_{x\in C_{i}}\;{\left\|x-\mu_{i}\right\|}^{2}\;. \end{array}$$ (20)

Here, *x* are the data points, *C*_1_, …, *C*_*k*_ are the *k* disjoint clusters and *μ*_*i*_ is the centroid of the *i*th cluster. We varied *k* and visually inspected the clustering. For consistency, we also clustered the data using other algorithms such as mean shift, spectral clustering and density-based spatial clustering of applications with noise. While there were minor differences between the suggested clusters, the overall clustering structure remained the same.

### Determining Ca^2+^ ISI distribution

To see which is the most likely ISI statistics, we apply the following protocol to every single cell from the experiment shown in Fig 1:

1. Choose one of the candidate ISIs (IP, IG, IIG) and estimate the most likely intensity function x*(*t*) using Bayesian inference as described above.
2. Compute the conditional intensity function based on x*(*t*), *q*(*t*|*y*_*k*_, x*), from Eq (15), and use it to plot the quantile-quantile and Kolmogorov-Smirnov diagrams.

### Performance of Ca^2+^ spike rate estimation

To test the performance of Ca^2+^ spike rate estimation, we generated surrogate data from an IG for the two different intensity functions *x*_det_(*t*) and *x*_GP_(*t*) using inverse sampling, a Bernoulli process based on the conditional intensity function in Eq (15) and time rescaling [47, 65, 66].

A key factor in estimating Ca^2+^ spike rates from PSTHs and KS is the choice of a bin width and bandwidth, respectively. For a large number of Ca^2+^ spike sequences, optimal estimates exist [49–51], and we use them for Fig 4. In case of only a few Ca^2+^ spike sequences with a small number of spikes per sequence, as in Fig 5, no estimates for a bin width or bandwidth exist. We therefore employed a bandwidth that was approximately equal to the optimal bandwidth determined in Fig 4 as well as bandwidths 1.5 and 2 times larger than this. In addition, we used the same formal expression as for the optimal value, which resulted in the bandwidth $\hat{\sigma }$. Note that $\hat{\sigma }$ differs from the optimal bandwidth in Fig 4, since it explicitly depends on the number of Ca^2+^ spike sequences.

### Calcium imaging of cells during dynamic stimulation

A bespoke perfusion system connected to a 3-port microfluidics device [67] was used to expose cultured HEK293T cells to varying concentrations of the muscarinic receptor agonist, carbachol. The HEK293T cell line was a gift from Dr N. Holliday, University of Nottingham, that had been frozen after passage 28 of the original stock. After thawing, cells were used for up to a further ten passages. Cells were seeded at a density of 10^5^ cells/ml in the central micro-channel of the microfluidic devices, in DMEM D6429 growth media (Invitrogen, Paisley, UK) containing 10% fetal calf serum. Cells were loaded inside the microchannels with 1 *μ*M of the Ca^2+^ indicator Fluo5F-AM for 30 min, followed by washout with imaging buffer (135 mM NaCl, 3 mM KCl, 10 mM HEPES, 15 mM D-glucose, 2 mM MgSO_4_ and 2 mM CaCl_2_) for at least a further 30 min. To stimulate the cells, the flow rates of two inlet channels into the microchannel were varied, allowing the interface between the two solutions to be shifted laterally across the chamber. One inlet stream contained the agonist (100 *μ*M carbachol) and Alexa Fluor 594 (AF594, 2 nM; to allow monitoring of agonist concentration in proportion to AF594 fluorescence). The second inlet contained buffer alone. The interface formed between the two solutions due to laminar flow was shifted across the width of the microchannel by controlled changes in the fractional flow rates for each stream, with total flow being constant. In combination with the shifting interface position, the concentration gradient formed by diffusional collapse of the interface as the co-flow progresses through the channel length results in a spatiotemporal gradient in agonist concentration throughout the channel. This method enables the exposure of cells to pre-defined, time-varying changes in agonist concentration, from simple step-changes to complex waveforms. During dynamic stimulation with agonist, AF594 and Fluo-5F AM indicators were excited sequentially (100 ms exposure, 1 Hz frame rate) using a pE2 LED system (excitation peaks 470 nm and 565 nm; CoolLED, Andover UK). Emission was detected at 535 ± 50 nm and 565 ± 20 nm with an ORCA-R^2^ camera (Hamamatsu, Welwyn Garden City, UK).

### Image analysis

A time-series analyser plugin to ImageJ (Wayne Rasband, National Institutes of Health, Bethesda, MD, available at http://rsb.info.nih.gov/ij) was used to manually define circular regions of interest (ROI) centred on each cell. Mean Fluo-5F emission intensity of pixels falling within each ROI was quantified and expressed as the ratio of fluorescence at time t divided by mean intensity from a 25 s window prior to the first increase in stimulus concentration (F/F_0_). The baseline window is selected as the window with minimum standard deviation from sliding 25 s windows taken from 0 to 120 s (before increase in stimulus concentration). Fluorescence of AF594 was quantified as the mean fluorescence intensity of pixels falling within each ROI being quantified; therefore each cell has a Ca^2+^ response measure and an associated stimulation profile.

## Supporting information

S1 Fig Determining Ca^2+^ spike ISI statistics. Quantile-quantile plots for Ca^2+^ spike sequences in HEK293T cells stimulated with (A) 100*μ*M and (B) 10*μ*M carbachol when the ISI statistics is assumed to be an IIG (blue), IP (red) and IG (grey) distribution. Box and whisker plots summarising the the quantile-quantile plots for (C) 100*μ*M and (D) 10*μ*M carbachol stimulation for the IP, IIG and IG models. The results in (A) and (C) are based on the data shown in Fig 1. The box extends from the first quartile (Q1) to the third quartile (Q3) with the red line at the median. The lower whisker corresponds to the smallest data point that is bigger than Q1−1.5×IQR, while the upper whisker extends to the largest value that is smaller than Q3+1.5×IQR, where IQR denotes the interquartile range Q3-Q1. We used 42 cells in (A), (C) and 21 cells in (B), (D).
(EPS)

S2 FigCa^2+^ spike rate estimation.Ca^2+^ spike rate estimations for data shown in Fig 4A for 1 (A), 2 (B), 4 (C) and 7 (D) randomly chosen Ca^2+^ spike sequences (black dots) using KS with *σ* = 35 (grey), 52 (orange), 70 (purple), $\hat{\sigma }$ (blue) and a GP(black). The dotted red line denotes the original Ca^2+^ spike rate. The light blue areas delineate the 95% GP estimation confidence interval.(EPS)

S3 Fig Statistical analysis of Ca^2+^ spike rate estimation for x_det_. Ca^2+^ spike sequences were generated using inverse sampling (A), a Bernouilli process (B) and time rescaling (C), and we computed the *L*_2_ norm between the true Ca^2+^ spike rate and the estimated Ca^2+^ spike rate based on the methods shown along the x-axis in each panel. For details of the box plot, see Fig 2. *N* = 100 for all three panels.
(EPS)

S4 Fig Statistical analysis of Ca^2+^ spike rate estimation for x_GP_. Ca^2+^ spike sequences were generated using inverse sampling (A), a Bernouilli process (B) and time rescaling (C), and we computed the *L*_2_ norm between the true Ca^2+^ spike rate and the estimated Ca^2+^ spike rate based on the methods shown along the x-axis in each panel. For details of the box plot, see Fig 2. We generated 100 calcium spike sequences with each generator for the statistical analsysis.
(EPS)

S1 Table Significant test for Ca^2+^ spike rate estimation. Based on the data reported in S3 and S4 Figs, we computed *p* values using the non-parametric Mann-Whitney test. Significant results (*p* < 5 × 10^−2^) are in bold.
(PDF)

S1 Video Stimulated HEK293T cells. HEK293T cells cultured in microfluidics chamber and loaded with Fluo-5F AM indicator. The green channel is Fluo-5F fluorescence emission, and the red channel is AF594 fluorescence emission. Time stamp is in seconds (frame rate of movie 20 f/s). Note delivery of carbachol (in same solution as AF594) at 150 s, by displacement of solution interface during laminar flow. This cell population is the same as shown in Fig 1 of the manuscript.
(MP4)

S1 Appendix Laplace’s integral approximation. (PDF)
