# Supplementary material for: A Bayesian approach to modelling heterogeneous calcium responses in cell populations
Source: PLoS Comput Biol. 2017 Oct 6;13(10):e1005794. doi: 10.1371/journal.pcbi.1005794 (PMC5646906; doi:10.1371/journal.pcbi.1005794)
Supplement: S1 Table — Based on the data reported in S3 and S4 Figs, we computed p values using the non-parametric Mann-Whitney test. Significant results (p < 5 × 10^−2^) are in bold. (PDF) [file pcbi.1005794.s005.pdf]

			KS $\hat{\sigma}$ vs GP	KS 35.0 vs GP	KS 52.0 vs GP	KS 70.0 vs GP
x_{det}	inverse sampling	p	$\mathbf{3.16 \times 10^{-34}}$	$\mathbf{7.80 \times 10^{-33}}$	$\mathbf{9.78 \times 10^{-14}}$	$\mathbf{7.49 \times 10^{-3}}$
	Bernoulli process	p	$\mathbf{2.98 \times 10^{-34}}$	$\mathbf{8.27 \times 10^{-33}}$	$\mathbf{3.72 \times 10^{-13}}$	$\mathbf{3.75 \times 10^{-2}}$
	time rescaling	p	$\mathbf{3.16 \times 10^{-34}}$	$\mathbf{2.99 \times 10^{-32}}$	$\mathbf{1.36 \times 10^{-15}}$	$\mathbf{2.79 \times 10^{-3}}$
x_{GP}	inverse sampling	p	$\mathbf{2.47 \times 10^{-27}}$	$\mathbf{1.55 \times 10^{-14}}$	2.40×10^{-1}	9.78×10^{-1}
	Bernoulli process	p	$\mathbf{3.32 \times 10^{-27}}$	$\mathbf{4.55 \times 10^{-16}}$	$\mathbf{1.10 \times 10^{-2}}$	2.83×10^{-1}
	time rescaling	p	$\mathbf{1.74 \times 10^{-29}}$	$\mathbf{5.08 \times 10^{-15}}$	1.37×10^{-1}	7.35×10^{-1}
