# Supplementary material for: A Bayesian approach to modelling heterogeneous calcium responses in cell populations
Source: PLoS Comput Biol. 2017 Oct 6;13(10):e1005794. doi: 10.1371/journal.pcbi.1005794 (PMC5646906; doi:10.1371/journal.pcbi.1005794)
Supplement: S1 Appendix — (PDF) [file pcbi.1005794.s007.pdf]

Laplace's integral approximation. We here show how to evaluate the integral $\int p(\mathbf{y}|x,\theta)p(x|\theta)\mathrm{d}x$ using Laplace's approximation. Consider a function $f:\mathbb{R}^n \to \mathbb{R}$ with a single maximum at x^*. Taylor expanding f around x^* results in

$$f(x) = f(x^*) + \frac{1}{2}(x-x^*)^T \mathcal{L}_x^2 f(x^*)(x-x^*) + O((x-x^*)^3)\,, \tag{1}$$

since $\nabla_x f(x^*) = 0$. When introducing the matrix $A = -\mathcal{L}_x^2 f(x^*)$, which is symmetric and positive definite, we find from Eq. (1)

$$\int_x \mathrm{e}^{f(x)}\mathrm{d}x \approx \mathrm{e}^{f(x^*)} \int_x \mathrm{e}^{-\frac{1}{2}(x-x^*)^T A (x-x^*)}\mathrm{d}x. \tag{2}$$

The integral on the right hand side of Eq. (2) is Gaussian, so that

$$\int_x \mathrm{e}^{f(x)}\mathrm{d}x \approx \mathrm{e}^{f(x^*)} \sqrt{\frac{(2\pi)^n}{\det(A)}}\,. \tag{3}$$

When we set $f(x) = \log p(\mathbf{y}|x,\theta)p(x|\theta)$ and note that

$$\mathcal{L}_x^2 f(x) = \mathcal{L}_x^2 \log p(\mathbf{y}|x,\theta) - \Sigma^{-1}\,, \tag{4}$$

since $p(x|\theta)$ is a GP with covariance function Σ, we arrive at

$$\int p(\mathbf{y}|x,\theta)p(x|\theta)\mathrm{d}x \approx p(y|x^*,\theta)p(x^*|\theta)\sqrt{\frac{(2\pi)^n}{\det(\Lambda^* + \Sigma^{-1})}}. \tag{5}$$

with $\Lambda^* = -\mathcal{L}_x^2 p(y|x^*,\theta)$.
